# Effect of Silane Coupling Agent on Modification of Areca Fiber/Natural Latex

**DOI:** 10.3390/ma13214896

**Published:** 2020-10-31

**Authors:** Yiren Pan, Meng Zhang, Jian Zhang, Xiaoyao Zhu, Huiguang Bian, Chuansheng Wang

**Affiliations:** College of Electromechanical Engineering, Qingdao University of Science and Technology, Qingdao 266061, China; pyr@qust.edu.cn (Y.P.); zhangmeng96710@163.com (M.Z.); Zhangjian10307533@163.com (J.Z.); 24833@woer.com (X.Z.)

**Keywords:** areca fiber, natural latex, silane coupling agent

## Abstract

In this paper, the areca fiber was extracted by physical and chemical treatment, and then the areca fiber/natural latex composite was prepared by natural latex impregnation technology. In order to combine areca fiber and natural rubber latex better, three silane coupling agents with different action mechanism were selected: Si−69, KH550, and KH570 which were used to treat the areca fiber/natural latex compound. The results show that the silane coupling agent can change the surface of the fiber from hydrophilic surface to organophilic surface, making the bonding of areca fiber to natural latex more closely. At the same time, the mechanical properties, physical and mechanical properties, swelling properties, and dynamic viscoelastic properties of the tightly bonded areca fiber/nature latex composites were improved. After observing the micro-structure through a scanning electron microscope, it was found that the three silane coupling agents could effectively bind areca fiber and natural latex to enhance the performance of the composite material, of which Si−69 performed best, and the tensile strength and tear strength of the composite increased by 21.19% and 12.90% respectively.

## 1. Introduction

With the development and research of rubber, it was found that the combination of natural rubber and fiber helps rubber heighten its properties of high modulus, high hardness, cutting resistance, tear resistance, puncture resistance, fatigue resistance, and creep resistance [1,2,3]. Natural plant fiber with its green environmental protection, renewable, degradable, pollution-free, and many other advantages has been widely used in medicine, construction, paper, textile, and other fields, and is becoming more and more mature [4]. Among them, areca as a tropical plant contains a lot of plant fiber, which can be purified by chemical methods to prepare a large number of areca fiber with good mechanical properties. For the rubber industry, the green treatment of waste tires and recycling of plant fiber is an urgent problem to be solved. In the past research, the waste tire powder was combined with ordinary plant fiber and the tire was reinforced by natural plant fiber, which could be used in plate production, which can improve the recycling of waste tire [5]. Since fiber and rubber are mainly bonded by physical friction and there are hydroxyl groups on the surface, the binding ability between them is poor. In order to give full play to the function of fiber, it is necessary to improve the interface adhesion between fiber and rubber through modification.

It is well known that one of the functions of silane coupling agent is as a surface modifier, which can effectively bond organic polymer and inorganic fillers. The inorganic filler is treated with silane coupling agent, changing the hydrophilic surface to the organophilic surface, which can not only avoid the agglomeration of particles in the system and the rapid thickening of the polymer, but also improve the mutual wettability of the organic polymer and reinforcement filler [6,7]. Furthermore, the carbon-functional silane can also make the reinforcing filler and polymer firmly bond. In previous studies, various coupling mechanisms have been proposed based on the reaction state, infiltration effect, and reaction conditions of silane coupling agents, among which the chemical bonding mechanism has been widely accepted. In terms of rubber reinforcement, the mechanism of action is as follows: The molecular structure of silane coupling agent is R–Si(OR′)_3_, where –R is the coupling reaction of organic functional group and organic substance and Si(OR′)_3_ is a silyloxy group, which can generally react with inorganic substances. During the reaction, the siloxane group would hydrolyze and generate –SiOH, and the oligosiloxane would form through the dehydration and condensation between –SiOH. Low polysiloxane contains a large number of unreacted –SiOH, and these unreacted siloxane oxygen groups form intermolecular hydrogen bonds with hydroxyl –OH on the surface of inorganic materials, forming a preliminary combination. In the subsequent mixing process, the silane coupling agent and hydroxyl –OH on the surface of the inorganic material will dehydrate to form a covalent bond, and complete the connection between the coupling agent and surface of the material in the form of molecular chains.

**Figure 1:**
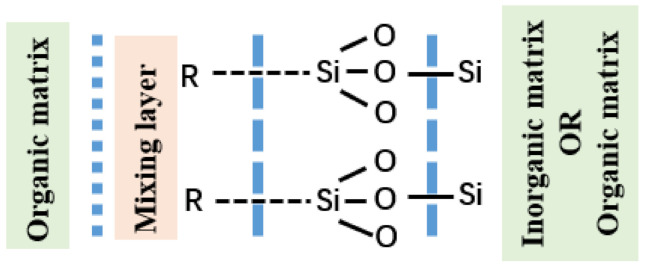
The coupling effect of silane coupling agent.

At the same time, the R group of the silane coupling agent diffuses into organic matter and infiltrated, forming a mixed layer of mutual doping between the silane coupling agent and organic matter, as shown in Figure 1. In the mixed layer, R groups interact with some active functional groups in organic compounds to form covalent bonds or hydrogen bonds that are the connected silane coupling agent and organic compounds. In previous studies, it was found that different silane coupling agents had different infiltration effects with organic compounds, and appropriate silane coupling agents should be selected in order to achieve the best modification effect.

In this paper, we mainly study the modified areca fiber as reinforcing material added to the natural latex, through experiments to explore the effects of areca fiber modified by different silane coupling agents on the properties of areca fiber/natural latex compound, and to find the optimum silane coupling agent that have the best modification effect on areca fiber.

## 2. Materials and Methods

### 2.1. Materials

Areca fiber: Fresh areca fiber, which was sold on the market. Natural latex: Total solid content of 60%, pH value about 10.0, Thailand; carbon black N330: Cabot (Tianjin, China) Corporation Co., Ltd. (Tianjin, China).; double-[γ-(triethoxysilyl)propyl] tetrasulfide Si−69 ([(C_2_H_5_O)_3_ SiCH_2_CH_2_CH_2_]_2_S_4_): Potassium hydroxide of Lianyungang Ruiba Chemical Co., Ltd. (Lianyungang, China); γ-aminopropyltriethoxysilane KH−550 (NH_2_C_3_H_6_Si(OC_2_H_5_)_3_: Jiangsu Chenguang Coupling Agent Co., Ltd. (Jiujiang City, China) NaOH; γ-methacryloxypropyltrimethoxysilane KH−570 (CH_2_=C(CH_3_)COOC_3_H_6_Si(OCH_3_)_3_)): Jiangsu Chenguang Coupling Agent Co., Ltd. (Jiujiang City, China); Sinopharm Group Chemical Reagent Co., Ltd. (Shanghai, China); hexamethoxymethylmelamine (tackifying resin) RA−65: Jiangsu Guoli Chemical Technology Co., Ltd.; Zinc Oxide ZnO: Aladdin Reagent Co. Ltd. (Shanghai, China); stearic acid SAD, Antioxidant RD (poly(1,2-dihydro-2,2,4-trimethyl-quinoline), Accelerator CZ (N-cyclohexylbenzothiazole-2-sulphenamide), and sulfur S are commercially available industrial grade products.

### 2.2. Experiment Formulation

The formula used in this study is shown in Table 1.

### 2.3. Preparation of Areca Fiber by Hot Alkali Method

**Figure 2:**
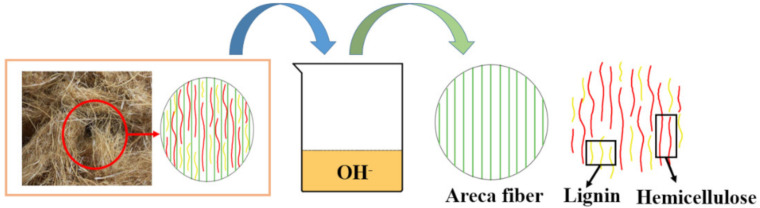
Preparation mechanism of fiber by alkali treatment.

Fresh areca fiber was peeled and the nucleolus was removed, before being crushed in a universal grinder for 4 h to obtain long fibrous areca fibers. The long fibers were washed with deionized water and dried for 7 h at 60 °C in a blast drying oven. After that, NaOH with a concentration of 14% was added and cooked at 140 °C for 4 h [8], with a liquid-solid ratio of 20:1. The cooked long fiber was washed with deionized water and dried again at 80 °C for 7 h to prepare the areca fiber. The process is shown in Figure 2.

### 2.4. Pretreatment of Silane Coupling Agent

1. Since Si−69 and KH570 are insoluble in water, it was necessary to prepare acetic acid with a concentration of 0.1%, before slowly adding the silane coupling agent and agitated aqueous solution quickly to prevent gel formation [9]. After dripping, the solution was shaken by ultrasound for 10 min until it was completely transparent. If impurities occurred, the solution would be filtered with a 0.5 sieve;
2. KH550 is an amino functional silane, which can be directly dissolved in water to prepare 1% aqueous solution, before being placed through ultrasonic vibration for 10 min to ensure uniform dispersion.

### 2.5. Modification Principle of Silane Coupling Agent

The main reason for choosing these three silane coupling agents among many silane coupling agents is the different action modes of these three silane coupling agents: Si−69 (bis-[γ-(triethoxysilyl)propyl] tetrasulfide) contains polysulfide bonds [10], which can increase the cross-linking bonds in the vulcanizate and increase the cross-linking density of the rubber; KH−550 (γ-aminopropyltriethoxysilane) is an alkaline coupling agent can effectively promote vulcanization and shorten the vulcanization time; and KH−570 (γ-methacryloxypropyl trimethoxysilane) contains C–C double bonds [11]. During vulcanization, the C–C double bonds can open to increase the cross-linking bond and increase the cross-linking density.

### 2.6. Preparation of Areca Fiber Latex Composite

**Figure 3:**
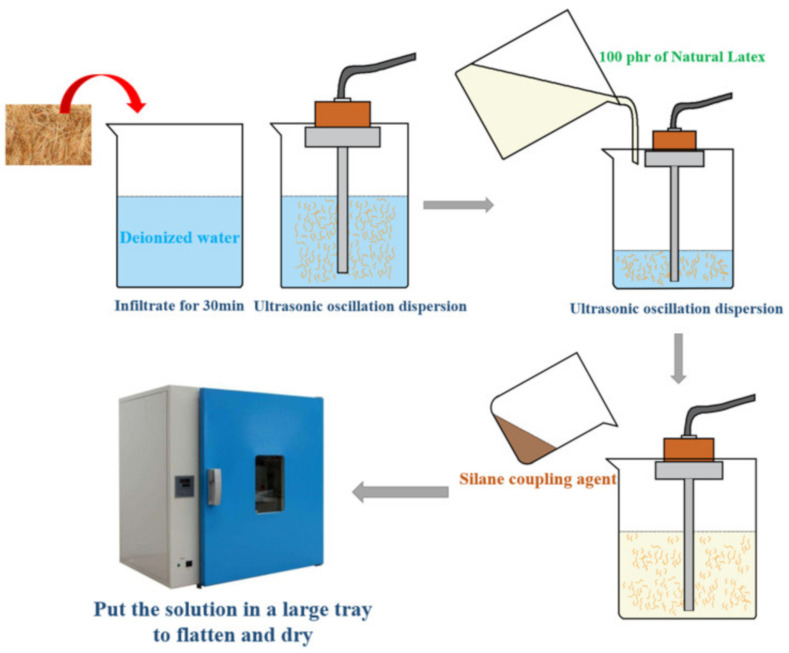
Process of areca fiber/natural latex compound.

The preparation process of areca fiber latex composite (Table 2) and the specific operation flow was shown in Figure 3.

### 2.7. Experimental Process

A rubber processing analyzer (RPA 2000, Alpha Technologies Akron, OH, USA) was used to test the dynamic rheological properties of the four systems. The strain scanning of the mixing rubber was as follows: Frequency of 1 Hz, temperature at 60 °C, and strain range at 0.1 °C. For rubber hardness, the determination of indentation hardness was done by means of a durometer (shore hardness). The dynamic strain was measured by dynamic mechanical analysis (DMA; GABOMETER−150, (GABO, Ahlden, Germany)). The tensile mode was used in temperature scanning with the frequency at 10 Hz and the heating rate at 2 °C/min. Tensile properties were tested using the UT-2060 tensile force testing machine produced by Taiwan U-CAN Technology (U-CAN, Taichung City, Taiwan). For scanning electron microscope (SEM, SU8000, Hitachi, Japan), the SEM acceleration voltage was 0.1–30kV. Fourier transform infrared spectrometer was used with Thermofisher Scientific Nicolet IS10 (PittCon (Waltham, MA, USA). Fourier transform infrared spectroscopy (FTIR) was used to test the possible chemical bonding in areca fiber before and after treatment. About 1 g of areca fiber samples were taken for each test.

Some parameters will be used in the analysis of experimental data, which are defined as follows. T10 can represent the scorch time, the time required from the start of heating to the increase of the feeding torque from high to low by 10 units. T90 positive curing process time is the time required from the beginning of heating to the torque of rubber compound increased by 90 units from low value. MH is the highest torque value, which can be used as a reference for the properties of rubber products and can also be used to measure the tensile strength and crosslinking density. ML is the minimum torque value and can be used as a reference for machining characteristics. – is the difference between the maximum torque the minimum torque, which can represent the crosslinking density.

### 2.8. Swelling Property of Adhesive

There is a crosslinked network in the crosslinked polymer and the polymer cannot be dissolved due to the presence of the crosslinked network. Due to the existence of water molecules in the polymer voids, the interaction between polymers was reduced (van der Waals force) for the crosslinked polymer, only to a certain degree of swelling, no matter how long it was in contact with the solvent, and the inhalation of the solvent quantity no longer increased and achieved balance, with the system in a constant two-phase state [13,14]. According to the mass or volume of the solvent absorbed by the polymer, the mass swelling degree Qm and volume swelling degree Qv are defined as follows:

(1) $$Q_{M}=\frac{M-M_{0}}{M_{0}}\;\mathrm{or}\;Q_{V}=\frac{V-V_{0}}{V_{0}}$$

where, M_0_ and V_0_ are the mass and volume of the sample before swelling, and M and V are the mass and volume of the sample during the swelling process. In addition, the swelling ratio q is defined:

(2) $$q=\frac{V}{V_{0}}$$

(3) $$Q_{V}=q-1.$$

## 3. Results

### 3.1. Chemical Bonding Analysis of Areca Fiber Prepared By Hot Alkaline Method

**Figure 4:**
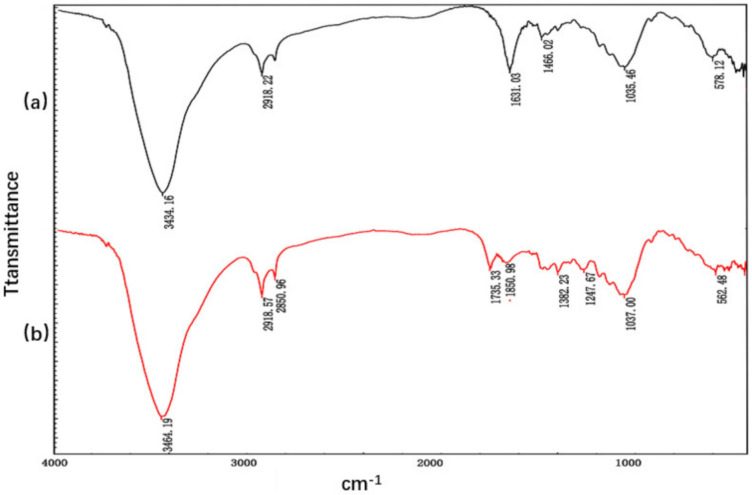
Infrared spectrum of areca fiber (**a**) before treatment and (**b**) after treatment.

Fourier infrared spectroscopy (FTIR) was used to analyze the possible and existing chemical bonds in areca fiber before and after treatment. Figure 4a shows the areca fiber before treatment, and Figure 4b shows the areca fiber after treatment with NaOH solution. As seen from Figure 4b, extensive strong peaks of areca fibers after NaOH solution treatment appeared at 3464 cm^−1^, indicating that more –OH groups existed in the alkali treatment process. After alkali treatment, the peak value of 1735 cm^−1^ disappeared, which can be seen from Figure 4b. The peak value was reduced to about 1730 cm^−1^, mainly due to the removal of acid, lignin, and other natural fiber components in this process. This result is consistent with previous studies by Sain and Pantha pulakkal [15] and Olaya [16]. It is worth noting that the peak value around 1730 cm^−1^ refers to the cross-linking of esters and ethers between cellulose and lignin, or cellulose and hemicellulose. After treatment with NaOH, hydrolysis occurred and ester or ether bonds were decomposed, resulting in the loss of the 1730 cm^−1^ peak. According to Mwaikambo and Ansell [17], peaks observed in the range of 1161–998 cm^−1^ indicate the presence of hemicellulose. In Figure 4b, a decrease in the peak strength of treated fibers is observed in this range, indicating that hemicellulose compounds are removed. Through the analysis of FTIR spectrum of areca nut fiber, it can be concluded that the more pure fiber can be effectively obtained by alkali treatment.

### 3.2. The Effects of Silane Coupling Agent on the Vulcanization Properties of Composite Materials

The effect of different silane coupling agents on the mechanical properties of composite materials is shown in Table 3. t_10_ was characterized by the scorch time of the compound. With the increase of scorch time, the early crosslinking phenomenon of linear molecules in the compound was weakened, and the possibility of premature vulcanization was reduced; t_90_ was the positive sulfuration time of the compound, and the crosslinking speed of linear molecules was accelerated, and the time to reach the maximum crosslinking density was reduced; and the crosslinking density of vulcanizates was reacted by MH-ML, and the greater the difference, the higher the crosslinking density [18]. It can be explained that the addition of silane coupling agent makes the vulcanization process close to the ideal state with increase of the vulcanization efficiency and crosslinking density. According to the analysis of the molecular structure of the three silane coupling agents and the principle of silane coupling reaction, KH−550 is an alkaline coupling agent, which can promote vulcanization in alkaline environment, so that the rubber compound can reach the maximum crosslinking density as soon as possible, and Si−69 and KH−570 contain multiple sulfur bonds and C–C double bonds. During vulcanization process of rubber, chemical bonds are opened and the maximum crosslinking density can be reached quickly by intensifying the crosslinking reaction. Furthermore, the addition of three silane coupling agents can avoid the premature vulcanization of the binder.

### 3.3. The Effects of Different Silane Coupling Agents on the Physical Properties of Compound

The effects of different silane coupling agents on the physical properties of the composite are shown in Table 4. It can be seen from the table that the addition of three silane coupling agents improves the constant elongation stress, tensile strength, tear strength, and abrasion performance of rubber samples. Mainly because −R groups in the silane coupling agent (organic functional groups) can react with natural rubber matrix, −Si(OR) siloxane reacts with the inorganic areca fiber and links organic matter (natural rubber) and organic matter (areca fiber), using the form of molecular bonds, which act as a bridge and forms a certain interface phase to make the binding between natural rubber and areca fiber closer.

At the same time, the existence of areca fiber hindered the movement of the molecular chain. The tighter the bond, the stronger the interface interaction, the greater the blocking effect, and the greater the stress required for the deformation of the composite sample. It can be seen from Table 4, that 100% constant elongation, 300% constant elongation, and tensile strength of the areca fiber/natural latex composite modified by the silane coupling agent are significantly higher than those of the unmodified areca fiber/natural latex composite. It can be fully explained that the areca fiber by the silane coupling agent had a stronger contact with rubber. It also fully indicates that under the high elongation strength, the stress will be transferred to the fiber through the well-bonded interface phase and the strength of the fiber itself will bear excess stress, playing a skeleton role in the composite material. At the same time, the abrasion of the modified areca fiber rubber composite decreased obviously [19].

Among the three silane coupling agents, Si−69 has the best effect for two possible reasons: First, each Si−69 (bis-[-(triethoxy silicon) propyl] tetrasulfide) monomer contains six ethoxy groups that can occur cross-aldol condensation more complete and faster. Secondly the group containing polysulfide bonds in the R group is easier to penetrate into the rubber matrix and has better compatibility with the rubber matrix, making the bonding more tight [20].

### 3.4. The Effects of Different Silane Coupling Agents on the Processing Performance of Areca Fiber/Natural Latex

**Figure 5:**
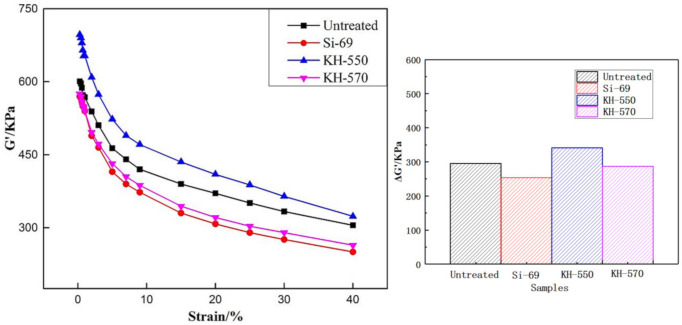
Rubber processing analyzer (RPA) curves of compound with different silane coupling agents.

Figure 5 shows the effect of different silane couplings on the swelling performance of vulcanizates. RPA curve represents the viscoelastic characteristics of the material in the process of dynamic deformation, which can represent the machinability of the material. The ordinate G’ represents the energy storage modulus of the sample and the abscissa is the size of the sample deformation. △G’ is the difference between large deformation and small deformation which can be used to characterize the Payne effect [21,22]. The larger △G’ indicates the stronger interaction between fillers, resulting in obvious agglomeration and poor dispersion of fillers, the smaller △G’ indicates that the dispersion of fillers was better and the network structure of the compound material was more uniform.

It can be seen from Figure 5 that the △G’ of the areca fiber/natural latex compound without the silane coupling agent is the largest, and the △G’ of the composite material with three kinds of silane coupling agent was smaller than that without the silane coupling agent, which indicates that adding the silane coupling agent can make the composite network structure more uniform. Among the three kinds of composites with the silane coupling agent, the composites with Si−69 have the smallest △G’ and the most uniform dispersion.

### 3.5. The Effects of Different Silane Coupling Agents on the Swelling Properties of Areca Fiber/Natural Latex

Swelling is a unique phenomenon of polymer. The size and motion velocity of the solvent molecules differ greatly from that of the polymer molecules. The diffusion velocity of the solvent molecules is fast while the diffusion of the polymer into the solvent is slow [23,24]. Therefore, the rubber volume will increase when the solvent molecules penetrate into the rubber compound. With the constant infiltration of solvent molecules, the volume of the swelling polymer material increases continuously and the movement of the macromolecular chain segment is enhanced. Then, through the coordinated movement of the chain segment, the movement of the entire macromolecular chain is achieved. Macromolecules gradually enter into the solution, forming a thermodynamically stable homogeneous system, namely, the dissolution stage. The movement of the whole macromolecular chain is achieved through the coordinated movement of the chain segments. The macromolecules gradually enter the solution and form a thermodynamic stable homogeneous system which is called the dissolution stage. When fiber is added into rubber, the interaction between them can be reflected in the swelling property of the composites. The smaller the swelling property of the composite, the stronger the interaction between fiber and rubber.

**Figure 6:**
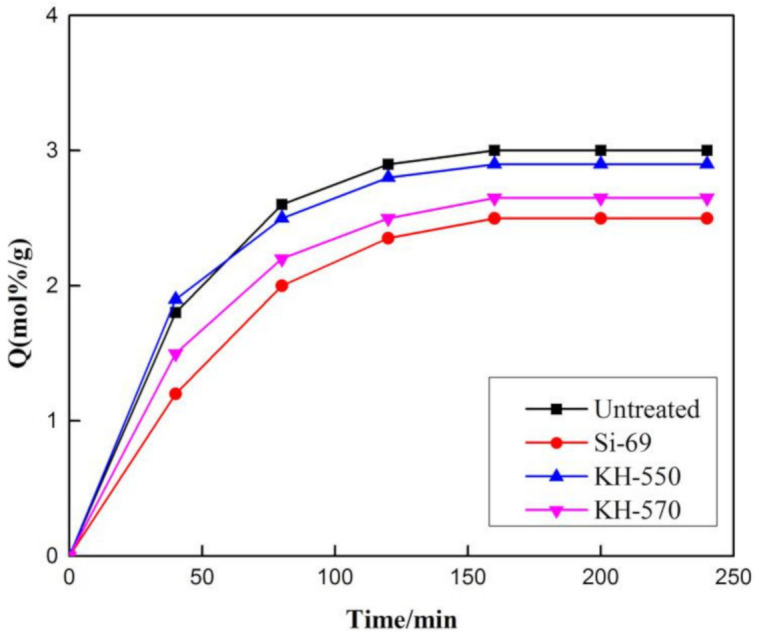
Effect of different silane coupling on swelling properties of vulcanizates.

It can be seen from Figure 6 that with the addition of the silane coupling agent, the solvent resistance of the natural latex/areca fiber composite was improved, among which Si−69 had the best effect, followed by KH−550. According to the swelling principle, we believe that the chemical bond formed by silane coupling reaction reduces the gap between the areca fiber and rubber matrix, areca fiber, and fillers. The crosslinking network may be more compact under the action of the silane coupling agent, according to the curve shown in Figure 6 the ability of solvent to enter the rubber matrix is weakened and the anti-swelling performance of composite material is improved. At the same time, the swelling coefficient of vulcanizate is also related to the crosslinking density of rubber. Generally, MH–ML can be used to compare the cross-linking density of rubber, shown in Table 3. It can be seen that the MH–ML value of the areca fiber/natural latex composite modified by Si−69 is the highest, indicating a higher the cross-linking density. However, Si−69 contains a large number of sulfur bonds, which can promote the vulcanization process and stabilize the cross-linking network. Therefore, the compound with the silane coupling agent Si−69 has the best swelling performance.

### 3.6. The Effects of Different Silane Coupling Agents on the Dynamic Mechanical Properties of Areca Fiber/Natural Latex

The effect of adding different silane coupling agents on the dynamic mechanical properties of vulcanizates is shown in Figure 8. The dynamic mechanical analysis (DMA) refers to the material in a composite mechanics response under the action of alternating force produced [25]. Tanδ is the ratio of the loss modulus E″ to the dynamic modulus E’ [26]. The higher the ratio, the higher the internal heat loss of the composite material will be when the external force does work on the compound. 

**Figure 7:**
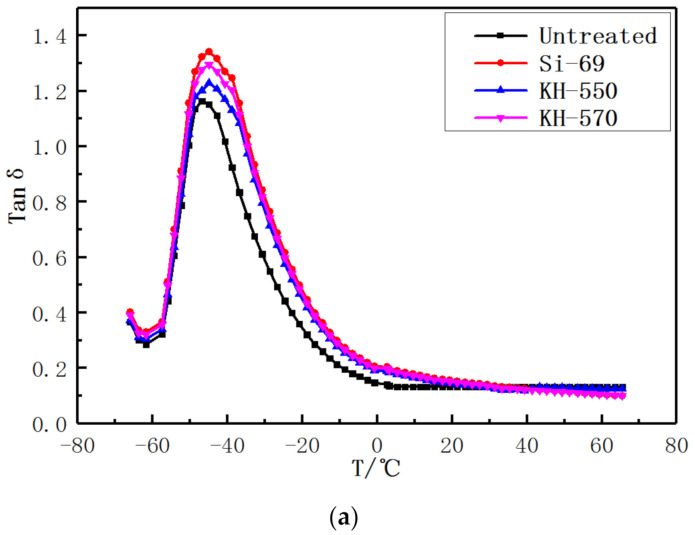

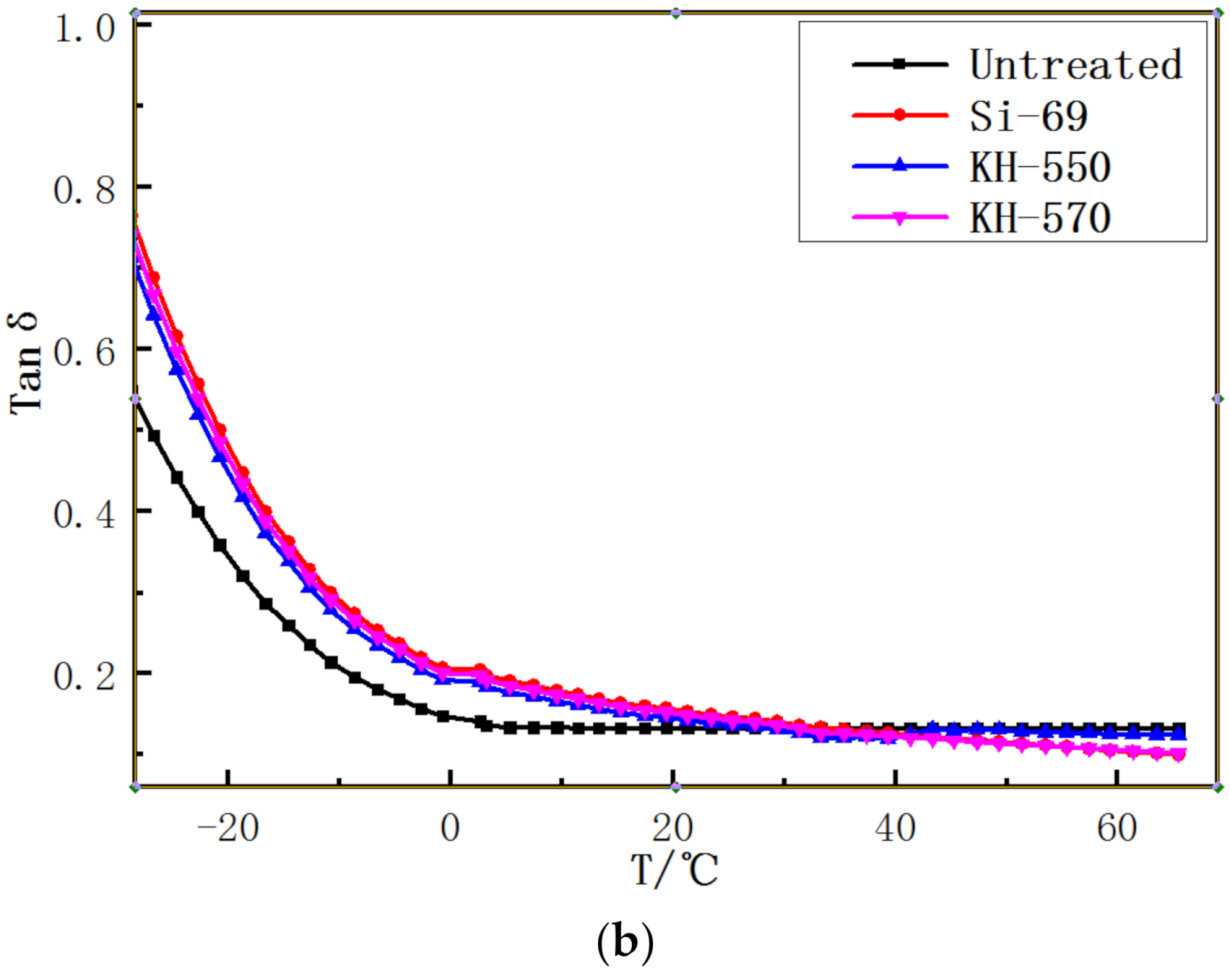
DMA curves of vulcanizates with different silane coupling agents (**a**) Dynamic mechanical analysis (DMA) and (**b**) DMA local amplification.

In Figure 7, the peak value of the curve Tan δ represents the glass transition temperature of the composites. When the temperature was lower than the glass transition temperature (Tan δ), the movement energy of the molecules in the composites is low, the polymer segments are difficult to move [27], and the fibers do not participate in the deformation. When the temperature exceeds the glass transition temperature (TG), the movement of the rubber molecular chain is intensified, and the fiber participates in the deformation process of polymer segment, which limits the movement of polymer chain segment. Therefore, when the fiber is well bonded to the rubber matrix, the chain segment activity decreases and Tg increases. It can be seen from Figure 7 that the Tg of the areca fiber/natural latex modified by Si−69 is higher than that of the other two silane coupling agents. Therefore, areca fiber modified by Si−69 can better combine with rubber matrix and play a higher reinforcing role.

At the same time, tan δ at 0 °C can represent the anti-wet sliding properties of rubber, and tan δ at 40 °C can represent the rolling resistance properties of rubber. According to the DMA data curve, tan δ at 0 °C of the areca fiber/natural latex composite modified by the silane coupling agent is higher than that of areca fiber/natural latex composite without modification, which indicates that the anti-wetting effect is good. At the same time, the tan δ at 40 °C of the modified areca fiber/natural rubber latex composite is lower than that of the unmodified areca fiber/natural latex composite, indicating high rolling resistance performance. In particular, the properties of the areca fiber/rubber composite modified by SI−69 improved significantly.

### 3.7. Effects of Different Silane Coupling Agents on the Microstructure of Areca Fiber/Natural Latex

**Figure 8:**
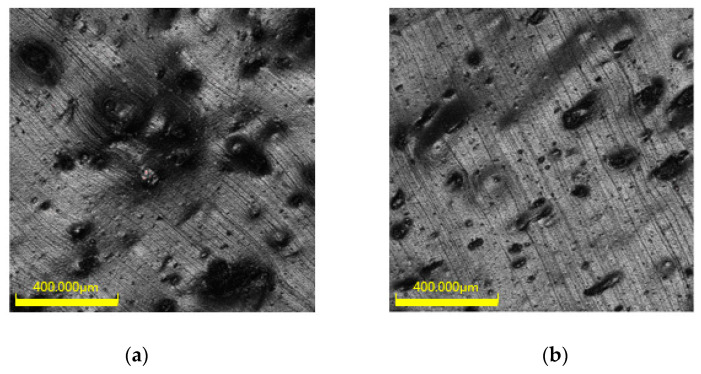

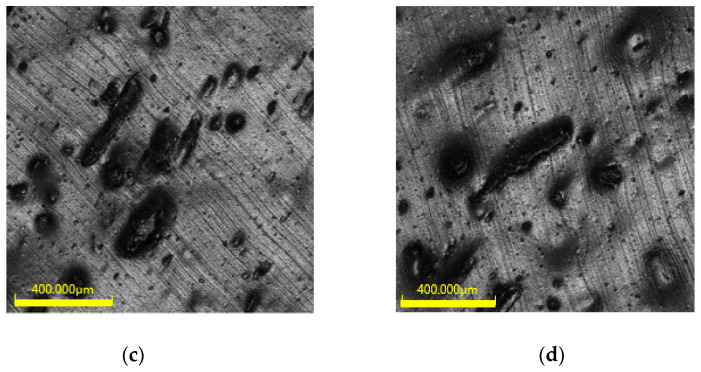
Images of areca fiber dispersion: (**a**) Untreated; (**b**) Si−69; (**c**) KH−550 and (**d**) KH−570.

**Figure 9:**
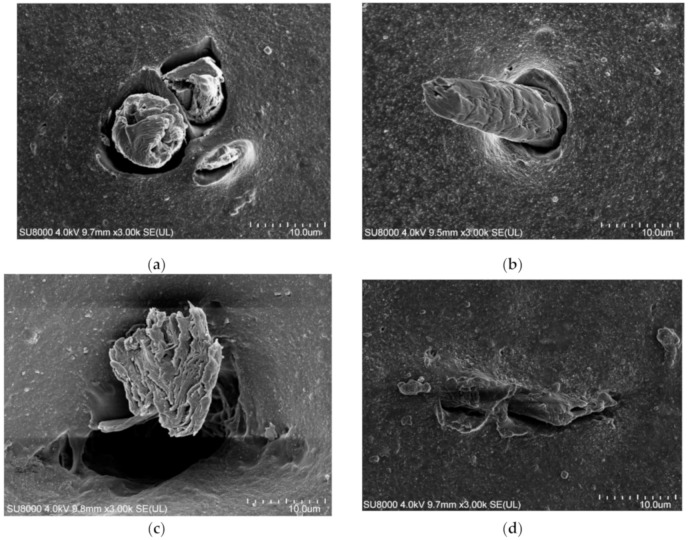
SEM of areca fiber/natural latex modified with silane coupling agent: (**a**) Untreated; (**b**) Si−69; (**c**) KH−550 and (**d**) KH−570.

Figure 8 shows the dispersion of fibers in rubber under Olympus three-dimensional morphology, and Figure 9 shows SEM photos of areca fiber/natural rubber latex modified by different silane coupling agents. According to Figure 8 and Figure 9, compared with the areca fiber modified by silane coupling agent, the areca fiber not modified by silane coupling agent had poor dispersion in rubber, and it is obvious that the results showed areca fiber agglomerated and had poor adhesion with rubber. By comparing the adhesive ability between rubber and areca fiber, which was modified by three silane coupling agents, it can be seen that the adhesive ability of the areca fiber modified by Si−69 is higher than that of areca fiber modified by other two silane coupling agents. At the same time, SEM images show that the areca fiber modified by Si−69 is more complete than the unmodified and other two silane coupling agents after the same preparation process and mixing process, which could improve the tensile strength and abrasion properties of rubber.

## 4. Conclusions

This paper mainly studied the effect of silane coupling agent on the modification of areca fiber/natural latex. According to the experimental results, it was found that the –R group in the silane coupling agent was an organic functional group which could react with organic compounds; −Si(OR′)_3_ was the silane oxygen group which could react with inorganic substance. Through these two reactions, areca fiber (inorganic substance) and rubber matrix (organic substance) were closely bonded to improve the performance of composite material. It could be seen from the swelling property of rubber composite that the solubility resistance of areca fiber/natural latex composite modified with silane coupling agent was better than that of areca fiber/natural latex composite modified without a silane coupling agent. It was obvious that the silane coupling agent could change the fiber surface from the hydrophilic surface to organophilic surface, which made the areca fiber bond more closely with the natural latex. In terms of mechanical properties and physical mechanical properties, the compound with the added Si−69 had the best performance, and its tensile strength and tear strength respectively increased by 21.19% and 12.90%. In terms of anti-slip performance and rolling resistance, the composite material with the Si−69 added was the best, and the composite material with the Si−69 added could be preferentially selected when the requirements for wet skid resistance and rolling resistance are high. Compared with the compound without the silane coupling agent, the compound with silane coupling agent had better mechanical properties, physical and mechanical properties, swelling properties, and dynamic viscoelastic energy. The silane coupling agent with the best performance could be selected according to the actual demand.

## Figures and Tables

**Table 1:** The experimental formula.

Material	Add Copies/phr	Material	Add Copies/phr
Natural latex	100	areca fiber	4
Carbon black N330	40	silane coupling agents	1.5
ZnO	5	RA−65	2.5
SAD	2	CZ	2
RD	2	S	2

**Table 2:** Preparation process of areca fiber latex composite.

List	Preparation Process
a.	Added 4 phr areca fibers were to deionized water for 30min. After infiltration, ultrasonic vibration was carried out until areca fibers were completely dispersed. Then 100 phr natural latex was added into the areca fiber and deionized water for several times, stirring constantly during the process to prevent the fiber from agglomeration; the prepared 1.5 phr silane coupling agent was poured into the mixed areca fiber/natural rubber latex mixture, and the ultrasonic oscillation was performed again, and the high-speed vibration would break the protective layer and gel layer of latex particles, increase the contact area between rubber hydrocarbon particles and areca fiber, and make the areca fiber evenly dispersed. At the same time, silane coupling agent in this process with fiber and latex in full contact, to achieve the bonding.
b.	Puted the mixed solution with silane coupling agent into a large tray and flatten it to dry. The drying temperature is 60 ℃ and the time is 8 h. Finally, puted areca fiber/natural latex master-batch into the mixer, and added fillers sequentially to prepare mixed rubber. After 8 h, it was vulcanized with a plate vulcanizing machine at 150 ℃ and 150 MPa. The vulcanized rubber was stored for 12 h for performance test [12].

**Table 3:** Effect of different silane coupling agents on the properties of mixed rubber.

Test List	Silane Coupling Agents			
	Untreated	Si−69	KH−550	KH−570
Mooney Viscosity [ML (1+4) 100 °C]	43	42	43	44
t10/min	1.55 ± 0.02	3.05 ± 0.04	3.55 ± 0.01	3.21 ± 0.03
t90/min	12.63 ± 0.06	9.25	8.33 ± 0.05	10.42 ± 0.03
ML (dN·m)	1.95 ± 0.54	1.55 ± 0.43	2.43 ± 0.57	1.65 ± 0.45
MH (dN·m)	15.47 ± 0.35	18.41 ± 0.26	16.13 ± 0.31	16.78 ± 0.28
MH–ML (dN·m)	13.52 ± 0.19	16.86 ± 0.17	13.7 ± 0.26	15.13 ± 0.17

**Table 4:** Effect of different silane coupling agents on the properties of vulcanizates.

Test List	Silane Coupling Agents			
	Untreated	Si−69	KH−550	KH−570
Hardness shore A/°	52	53	51	54
100% modulus/MPa	3.56 ± 1.42	4.45 ± 0.72	3.68 ± 0. 84	4.21 ± 0.78
300% modulus/MPa	14.21 ± 0.96	16.59 ± 0.85	15.21 ± 1.02	15.43 ± 1.15
Tensile strength/MPa	22.45 ± 1.34	27.21 ± 0.86	24.66 ± 1.21	23.32 ± 1.26
Elongation at break/%	320.3 ± 4.2	443.1 ± 2.8	394.2 ± 3.1	415.4 ± 2.3
Tear strength/KN·m^−1^	62 ± 2.5	70 ± 1.8	66 ± 2.3	68 ± 2.7
Abrasion loss/cm^3^	0.115	0.091	0.103	0.104
